# Association of the meaning of life with satisfaction, the occurrence of subjective complaints and the family’s economic status in the population of lower secondary school students

**DOI:** 10.34763/devperiodmed.20172101.6068

**Published:** 2017-05-29

**Authors:** Dorota Zawadzka, Magdalena Stalmach, Anna Oblacińska, Izabela Tabak

**Affiliations:** 1 Zakład Psychologii Klinicznej Dzieci i Młodzieży Akademii Pedagogiki Specjalnej im. Marii Grzegorzewskiej, Warszawa Poland; 2 Zakład Zdrowia Dzieci i Młodzieży Instytutu Matki i Dziecka, Warszawa Poland

**Keywords:** poczucie sensu życia, zadowolenie z życia, dolegliwości subiektywne, status ekonomiczny rodziny, meaning in life, satisfaction with life, subjective complaints, economic status of family

## Abstract

**Introduction:**

Feeling of meaning in life is extremely crucial factor of mental health. The lack of it can result in various disorders. Many authors, especially those connected with current of humanistic psychology underline the teenagers’ life sense.

**The aim:**

The aim of the paper was to examine the level of satisfaction with life, the frequency of psychosomatic complaints by junior high school students as well as the estimation of economical status of family and the analysis of meaning in life with above mentioned factors.

**Material and methods:**

The research was carried out in 2015 at 70 schools from all over the country, in group of 3695 lower secondary school students of I-III classes at the age of 13-17 (M=14,9; SD=0,87). The analysis connected with meaning in life using the shorten version of Purpose in Life Test (PIL) as well as analysis of life satisfaction using Cantril scale were taken up. What is more, the subjective physical complaints using single-factor shorten scale and economic status of family with the usage of material resources FAS scale (Family Affluence Scale) were examined. The statistical analysis included a one-way analysis of variance (ANOVA), t-student test post-hoc test as well as multivariate logistic regression model.

**Results:**

The average level of meaning in life among the examined students was 24,7 points (the summary scale 0-36 points), the boys achieved higher score than girls. The students satisfied with life (t=28,0; p<0,001), with rare physical complaints (F=124,8; p<0,001) and from affluent families (F=15,4; p<0,001) were significantly characterized by higher average level of meaning in life than students who were dissatisfied with their life, often or fairly suffer from health complaints and live in families of at most average level of affluence.

**Conclusion:**

The meaning in life is positively connected with satisfaction with life, lack of subjective complaints and family affluence. Because there is a lack of analysis linked with school teenagers’ meaning in life in Polish literature, another research involved not only shorten but also full version of this tool should be conducted.

## Streszczenie

**Wstęp:**

Poczucie sensu życia to niezwykle istotny wskaźnik zdrowia psychicznego, którego deficyt może prowadzić do wielu zaburzeń funkcjonowania psychospołecznego. Wielu autorów, zwłaszcza związanych z nurtem psychologii humanistycznej, podkreśla znaczenie sensu życia w kształtowaniu zdrowia nastolatków.

**Cel:**

Celem pracy było zbadanie poziomu poczucia sensu i zadowolenia z życia oraz częstości odczuwania dolegliwości subiektywnych przez uczniów gimnazjum, w odniesieniu do statusu ekonomicznego rodziny oraz analiza związku sensu życia z powyższymi zmiennymi.

**Materiał i metody:**

Badanie przeprowadzono w 2015 roku w 70 szkołach z terenu całego kraju, w grupie 3695 uczniów klas I-III gimnazjum w wieku 13-17 lat (M=14,9; SD=0,87). Podjęto analizy dotyczące poczucia sensu życia z zastosowaniem skróconej wersji skali Testu Sensu Życia − Purpose in Life Test (PIL), zadowolenia z życia przy użyciu skali Cantrila, występowania wybranych dolegliwości subiektywnych oraz statusu materialnego rodziny z zastosowaniem skali zasobów materialnych FAS − Family Affluence Scale. Analizy statystyczne obejmowały jednoczynnikową analizę wariancji (ANOVA), test t-Studenta, tzw. test post-hoc oraz wielowymiarowy model regresji logistycznej.

**Wyniki:**

Średni poziom sensu życia wśród badanych uczniów wyniósł 24,7 punktów (zakres sumaryczny: 0-36 punktów), chłopcy osiągnęli wyższą punktację niż dziewczęta. Uczniowie zadowoleni ze swojego życia (t=28,0; p<0,001), rzadko odczuwający dolegliwości subiektywne (F=124,8; p<0,001) oraz pochodzący z rodzin zamożnych (F=15,4; p<0,001), charakteryzowali się istotnie wyższym średnim poziomem sensu życiu niż uczniowie, którzy byli niezadowoleni ze swojego życia, przeciętnie i często odczuwali dolegliwości oraz pochodzili z rodzin o co najwyżej przeciętnym poziomie zamożności.

**Wnioski:**

Poczucie sensu życia jest pozytywnie związane z zadowoleniem z życia, brakiem odczuwania dolegliwości subiektywnych oraz zamożnością rodziny.

Ponieważ w literaturze polskiej brakuje analiz dotyczących poczucia sensu życia młodzieży szkolnej, warto przeprowadzić kolejne badania, z uwzględnieniem zarówno skróconej, jak i pełnej wersji Purpose in Life Test.

## Wprowadzenie

### Poczucie sensu życia

W istniejącej literaturze można znaleźć różny sposób definiowania pojęcia sensu życia. Na przykład, *Viktor Emil Frankl* (1992) w książce „Człowiek w poszukiwaniu sensu”, stwierdza, iż nie należy poszukiwać jakiegoś abstrakcyjnego sensu życia, gdyż każdy człowiek ma swoją misję, którą powinien wypełnić i w której ma szansę odnaleźć sens swego istnienia. Powyższa koncepcja skłania ku myśleniu, iż człowiek w każdym momencie może czuć potrzebę podjęcia trudu, którym jest odnalezienie oraz wypełnienie swojego życia sensem. Poszukiwanie sensu życia można więc uznać za podstawową motywację w życiu człowieka [1]. Sens życia nie stanowi łatwej, swoistej potrzeby pośród wielu innych potrzeb. Rozumiany jest tutaj jako połączenie skutków dążeń oraz wartości, które reprezentuje człowiek. Można postawić tezę, iż człowiek pyta o sens, odnosząc się do wartości, którymi kieruje się w życiu [2].

Poczucie sensu życia to niezwykle istotny wskaźnik zdrowia psychicznego, którego deficyt może prowadzić do wielu zaburzeń oraz braku motywacji do podejmowania trudów dnia codziennego. W konsekwencji może spowodować dużą obojętność, prowadzącą do rezygnacji z życia, zarówno u osoby dorosłej, jak i młodzieży [3, 4]. Wielu autorów, zwłaszcza związanych z nurtem psychologii humanistycznej, sygnalizuje znaczenie poczucia sensu życia u nastolatków [5, 6].

W okresie dojrzewania i dorastania pojawia się wskazywanie sobie odległych celów i podejmowanie intensywnych działań zmierzających do ich realizacji. Różnią się one w zależności od warunków, w jakich młodzież żyje. Formowane są nowe rodzaje motywacji, zmianie ulegają treści i organizacja przeżyć uczuciowych. Występujące w tym czasie trudności dotyczą m.in. nadania sensu własnemu życiu.

Poszukiwanie własnej tożsamości związanej z poczuciem sensu życia składa się, według K. Obuchowskiego [7] z trzech faz:

– Faza identyfikacji – utożsamianie się z zewnętrznymi wzorami.
– Faza kosmiczna – oderwanie od rzeczywistości, roz/mach, chaos w poszukiwaniu celu i sensu życia.
– Faza dojrzałego sensu życia (późna adolescencja i lata dalsze) – umiejętność określenia siebie i sensu swojego życia. Faza ta kształtuje się stopniowo, w okresie dojrzewania płciowego i dorastania.

### Zadowolenie z życia

Zadowolenie z życia, jako poznawczy komponent subiektywnego samopoczucia, zajmuje wiele uwagi naukowców w dziedzinie psychologii pozytywnej [8, 9, 10]. Niektórzy z nich podkreślają wpływające na zadowolenie z życia czynniki kontekstowe, takie jak: stosunki społeczne, dostępność zasobów materialnych i religijność [11]. Zadowolenie z życia jest określane jako subiektywna ocena poznawcza życia jednostki jako całości [12]. Zadowolenie z życia i szczęście są wskaźnikami subiektywnego samopoczucia, które jest jednym z głównych aspektów zdrowia psychicznego [13], ale także fizycznego. Ludzie zdrowi wydają się być bardziej usatysfakcjonowani i szczęśliwi niż ci, którzy cierpią na choroby fizyczne [14, 15].

### Dolegliwości subiektywne

Z pojęciem zdrowia subiektywnego, zaproponowanym przez C. Ry= [16], łączy się koncept dobrostanu (*well-being*), czyli dobrego samopoczucia, psychicznego i społecznego, które może być najlepiej określone przez daną osobę. Obszar zdrowia subiektywnego może zawierać szereg świadomych i nieświadomych elementów, np. poczucia „rezerwy” zdrowia czy poczucia niedoboru zdrowia lub dysfunkcji [17]. Znamiennym zaburzeniem stanu zdrowia w okresie dojrzewania są dolegliwości, których nie można wyjaśnić przyczynami organicznymi (*zycznymi). Obecnie określa się je jako dolegliwości subiektywne. W przypadku niektórych zaburzeń, składnik emocjonalny stanowi bezpośredni impuls powstawania problemu, w innych – jest jedynie elementem szeregu różnych uwarunkowań.

### Status ekonomiczny rodziny

Nierówności społeczne w zdrowiu są podstawowym elementem społecznych badań epidemiologicznych, a status socjoekonomiczny (SES) jest ściśle związany ze zdrowiem [18]. Znaczenie statusu socjoekonomicznego zostało omówione w wielu pracach i znaleziono potwierdzenie w wynikach badań, że FAS wiąże się zarówno z pozycją społeczno-gospodarczą (SEP), jak też różnymi wskaźnikami zdrowia [19, 20, 21].

Biorąc pod uwagę, że zadowolenie z życia, zdrowie subiektywne oraz status materialny rodziny są niezwykle ważnymi zmiennymi w badaniach dzieci i młodzieży szkolnej, zdecydowano się sprawdzić ich związek również z poczuciem sensu życia młodzieży w wieku 13-17 lat.

## Cel

Celem pracy było zbadanie poziomu sensu i zadowolenia z życia oraz częstości odczuwania dolegliwości subiektywnych przez uczniów gimnazjum, a także ocena statusu ekonomicznego rodziny. Podjęto również analizę związku między poczuciem sensu życia a pozostałymi zmiennymi kontekstowymi.

Postawiono dwa pytania badawcze:

1. Czy wymienione powyżej zmienne są różnicowane przez płeć i wiek uczniów?

2. Czy zadowolenie z życia, dolegliwości somatyczne i status ekonomiczny rodziny są związane z poczuciem sensu życia?

## Materiał i metoda

### Badane osoby i procedura badania

Praca przedstawia wyniki analiz prowadzonych z wykorzystaniem danych uzyskanych z ogólnopolskiego badania ankietowego w szkołach w 2015 roku, w ramach projektu NCN pt.: *„Środowisko fizyczne i społeczne oraz jakość funkcjonowania szkoły a zdrowie subiektywne i zachowania zdrowotne”* (Nr 2013/09/B/HS6/03438). Na przeprowadzenie badania uzyskano zgodę Komisji Bioetycznej przy Instytucie Matki i Dziecka.

Badaniem objęto grupę 3695 uczniów klas I-III gimnazjum w wieku 13-17 lat (1754 chłopców i 1941 dziewcząt), w 70 szkołach z terenu całego kraju, po uprzednim uzyskaniu zgody dyrektora szkoły oraz uczniów i ich rodziców. Źródło danych stanowi anonimowa ankieta audytoryjna pt. „Zdrowie i szkoła”, wypełniana przez uczniów w tradycyjnej formie papierowej lub on line.

### Badane zmienne i ich pomiar

Podjęte w pracy analizy dotyczyły następujących zagadnień:

1. **Poczucie sensu życia**, badano przy użyciu Testu Sensu Życia – Purpose in Life Test (PIL) autorstwa J. C. Crumbaugha i L. T. Maholicka, w polskiej adaptacji Z. Płużek [22]. W badaniu zastosowano skróconą, jednoczynnikową wersję testu, której analiza psychometryczna została przeprowadzona przez polskich badaczy pod kierunkiem J. Życińskiej [23] Składa się ona z 6 stwierdzeń ocenianych od 0 do 7 punktów, które odnoszą się bezpośrednio do posiadania celu oraz odnajdywania sensu i roli w życiu. Opracowano indeks sumaryczny przyjmujący zakres od 0 do 36 punktów. Generalnie im wyższy wynik uzyskany na skali, tym wyższym poziomem poczucia sensu życia charakteryzowała się badana młodzież. Na zastosowaną w badaniu wersję skali składają się następujące stwierdzenia:

- W życiu: nie mam żadnych celów ani do niczego nie dążę (1) − mam bardzo wyraźne cele i dążenia (7);
- Moje istnienie jest: zupełnie bezcelowe (1) – celowe i sensowne (7);
- Każdy dzień: niesie ze sobą coś nowego (7) – jest zawsze taki sam (1);
- W dążeniu do celów życiowych: nigdy nie miałem powodzenia (1) – udało mi się zaspokajać moje potrzeby (7);
- Uważam, że moje szanse na znalezienie sensu życia, celu i roli w życiu: są bardzo duże (7) − są praktycznie żadne (1);
- Doszedłem do wniosku, że: brak mi celu (1) − mam wyraźne cele dające pełne zadowolenie (7).

Dokładniejsze informacje dotyczące zastosowanej skali zostały przedstawione w odrębnym opracowaniu [24].

2. **Zadowolenie z życia**, badano z zastosowaniem skali wizualnej, będącej adaptacją tzw. skali Cantrila i stosowanej w badaniach HBSC (*Health Behaviour in School-aged Children)* od 2002 roku [25]. Skala ta przyjmuje zakres od 0 do 10 punktów. Do skali dołączona jest instrukcja:

Obok jest rysunek drabiny. Na górze drabiny jest liczba 10 – umownie oznaczająca życie, które wydaje Ci się najlepsze. Na dole drabiny jest liczba 0 – oznaczająca życie, które wydaje Ci się najgorsze. Pomyśl, jakie jest teraz Twoje życie i w którym miejscu drabiny Ty stanąłbyś. Wstaw X w jedną kratkę w drabinie cyfry, która znajduje się w tym miejscu.

Przyjęto podział dychotomiczny i założono że osoby, które wybrały poziom 0-5 punktów są niezadowolone ze swojego życia, natomiast te, które uzyskały 6-10 punktów to osoby zadowolone z życia.

3. **Dolegliwości subiektywne**, badano za pomocą skróconej, jednoczynnikowej skali będącej częścią skali cząstkowej *Problemy zdrowia fizycznego*. Skala ta wraz z dwoma innymi skalami cząstkowymi (*Problemy emocjonalne* i *Ograniczenia aktywności*) pochodzi z kwestionariusza CHIP-AE *(Child Health and Illness Profile: Adolescent Edition)* [26]. Do zastosowanej w naszych badaniach skróconej skali należą 3 stwierdzenia dotyczące częstości odczuwania dolegliwości somatycznych, oceniane na skali 5-stopniowej: *W ciągu ostatnich 4 tygodni, przez ile dni naprawdę źle się czułeś?; budziłeś się z uczuciem zmęczenia?; miałeś bóle głowy?* Pytanie

poprzedzone było instrukcją: *zaznacz znakiem X jedną kratkę w każdym wierszu*, a każde stwierdzenie miało kategorie odpowiedzi: *wcale; od 1 do 3 dni; od 4 do 6 dni; od 7 do 14 dni; od 15 do 28 dni*. Dla potrzeb analiz kategoriom tym przyporządkowano odpowiednio od 0 do 4 punktów. Odpowiedzi zostały wykorzystane do opracowania indeksu sumarycznego przyjmującego zakres od 0 do 12 punktów. Im wyższy wynik uzyskany na skali, tym częściej badana młodzież odczuwała dolegliwości somatyczne w ciągu ostatnich 4 tygodni. Dokonano podziału na trzy kategorie w zależności od częstości odczuwania dolegliwości somatycznych: rzadko (0-1 punkt); przeciętnie (2-6 punktów); często (7-12 punktów).

4. **Status materialny rodziny** oceniano z zastosowaniem skali zasobów materialnych - FAS (*Family Auence Scale*). Stopniowo modyfikowana skala FAS wykorzystywana jest w badaniach HBSC jako wiarygodny miernik statusu społecznoekonomicznego rodziny [27, 28]. W obecnym badaniu posłużono się trzecią wersją tej skali (FAS III), na którą składa się sześć pytań, które dotyczą: posiadania własnego pokoju przez ucznia, liczby samochodów w rodzinie, liczby komputerów w rodzinie, wyjazdów z rodziną na wakacje lub ferie za granicę, a także liczby łazienek w domu i wyposażenia gospodarstwa domowego w zmywarkę do naczyń. Zastosowana rozszerzona wersja skali FAS przyjmuje zakres od 0 do 13 punktów. Wyniki skali zostały ujęte w 4 kategorie rodzin: *biedne* (0-4 punkty), *raczej biedne* (5-6 punktów), *przeciętne* (7-9 punktów) i *zamożne* (10-13 punktów). Kategoryzacji dokonano zgodnie z krajowymi wytycznymi, uwzględniającymi duży odsetek rodzin z wynikiem w skali FAS poniżej 7 punktów [28].

## Analizy statystyczne

W pierwszej części pracy przedstawiono wyniki analiz jednowymiarowych, dotyczące średniego poziomu sensu życia, poziomu zadowolenia z życia oraz częstości odczuwania dolegliwości subiektywnych. Dalsze analizy prowadzono na zmiennych skategoryzowanych. Do zbadania różnic w poziomie poczucia sensu życia u uczniów w zależności od ich poziomu zadowolenia z życia oraz częstości odczuwania dolegliwości subiektywnych i statusu ekonomicznego rodziny zastosowano jednoczynnikową analizę wariancji (ANOVA), test t-Studenta oraz tzw. test posthoc czyli test porównań wielokrotnych. Analizy te są stosowane gdy analiza wariancji informuje, że są istotne statystycznie różnice jednak nie wiadomo, które z porównywanych grup różnią się miedzy sobą W celu określenia prawdopodobieństwa wysokiego poziomu sensu życia w kontekście wybranych zmiennych, oszacowano wielowymiarowy model regresji logistycznej, dzieląc umownie zmienną dotyczącą poczucia sensu życia na 2 kategorie: *wysoki poziom poczucia sensu życia* (powyżej 26 punktów) oraz *brak wysokiego poczucia sensu życia* (poniżej 26 punktów). Do modelu regresji włączono również płeć oraz klasę (od I do III) gimnazjum jako zmienne kontrolowane. We wszystkich analizach za poziom istotności przyjęto p<0,05. Zastosowano program SPSS v. 17.

## 1 Analiza jednowymiarowa

### 1.1 Średni poziom poczucia sensu i zadowolenie z życia oraz dolegliwości subiektywne

Tabela I prezentuje dane dotyczące średniego poziomu następujących zmiennych poczucia sensu życia, zadowolenia z życia oraz dolegliwości subiektywnych badanych uczniów.

**Tabela I:** Poczucie sensu życia oraz zadowolenie z życia i dolegliwości subiektywne (średnie i odchylenia standardowe). Table I. Meaning in life, life satisfaction and subjective complaints (mean and SD).

Średnie (odchylenie standardowe) *Mean value (SD)*	Ogółem *Total N=3695*
Poziom poczucia sensu życia (0-36p.) Level of meaning in life	24,7 (7,4)
Zadowolenie z życia (0-10p.) Satisfaction with life	7,03 (2,35)
Częstość odczuwania dolegliwości fizycznych (0-12p.) Subjective complaints	4,03 (3,13)

### 1.2 Poczucie sensu życia, zadowolenie z życia i dolegliwości subiektywne według płci i poziomu edukacji

W toku analizy zebranego materiału stwierdzono, że chłopcy charakteryzowali się istotnie wyższym poziomem sensu życia niż dziewczęta (p<0,001). Ustalono również, że klasa gimnazjum istotnie różnicuje średni poziom sensu życia uczniów (p<0,014). Analizując powyższe zmienne po skategoryzowaniu, odnotowano różnice w poziomie zadowolenia z życia w zależności od płci i poziomu edukacji (klasy gimnazjum). Różnice te były istotne statystycznie tylko w zależności od płci, przy czym chłopcy istotnie częściej niż dziewczęta byli zadowoleni z życia (p<0,001). Częstość odczuwania przez młodzież dolegliwości subiektywnych była różnicowana istotnie statystycznie zarówno przez płeć, jak i klasę gimnazjum (p<0,001). Dziewczęta oraz uczniowie III klasy gimnazjum istotnie częściej niż chłopcy i uczniowie młodsi deklarowali nasilone odczuwanie dolegliwości subiektywnych (tab. II).

**Tabela II:** Średni poziom poczucia sensu życia oraz zadowolenie z życia i dolegliwości subiektywne wg płci i klasy. Table II. Mean value of meaning in life, satisfaction with life and subjective complaints by gender and grade.

	Poczucie sensu życia, zadowolenie z życia i dolegliwości subiektywne *Meaning in life, satisfaction with life and subjective complaints*						
	Chłopcy *Boys* N=1754	Dziewczęta *Girls* N=1941	p	I klasa *Ist grade* N=1310	II klasa *IInd grade* N=1390	III klasa *IIIrd grade N=1250*	p
**Średni poziom poczucia sensu życia*Mean value of life meaning***							
Średnia (odchylenie standardowe) *Mean value (SD)*	25,1 (7,1)	24,3 (7,5)	**0,001**	25,2 (7,3)	24,4 (7,4)	24,4 (7,4)	**0,014**
**Zadowolenie z życia *Satisfaction with life***							
Tak *Yes*	78,9	73,1	<0,001	77,1	75,8	74,7	0,388
Nie No	21,1	26,9		22,9	24,2	25,3	
**Dolegliwości subiektywne *Subjective complaints***							
Rzadko ** odczuwane *Rarely felt*	33,7	17,0	**<0,001**	29,8	24,3	20,5	<0,001
Przeciętnie ** odczuwane *On average felt*	54,2	55,8		51,9	55,3	57,9	
Często ** odczuwane *Often felt*	12,2	27,2		18,3	20,3	21,6	

### 1.3 Sens życia a zadowolenie z życia, dolegliwości subiektywne u uczniów oraz status materialny rodziny

Analiza wariancji ANOVA oraz porównania wielokrotne wskazały na istotne statystycznie różnice w średnim poziomie sensu życia między uczniami w zależności od ich zadowolenia z życia i częstości odczuwania dolegliwości fizycznych oraz statusu ekonomicznego rodziny (ryc. 1). Uczniowie zadowoleni ze swojego życia (t=28,0; p<0,001), rzadko odczuwający dolegliwości fizyczne (F=124,8; p<0,001) oraz pochodzący z rodzin zamożnych (F=15,4; p<0,001), charakteryzowali się istotnie wyższym średnim poziomem sensu życia niż uczniowie, którzy byli niezadowoleni ze swojego życia, przeciętnie i często odczuwali dolegliwości oraz pochodzili z rodzin o najwyżej przeciętnym poziomie zamożności.

**Ryc. 1:**
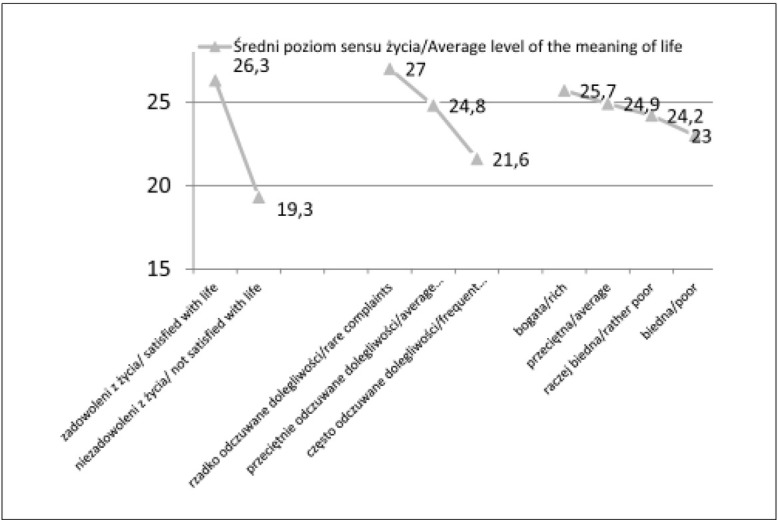
Średni poziom poczucia sensu życia uczniów gimnazjum w zależności od zadowolenia z życia i dolegliwości subiektywnych oraz statusu ekonomicznego rodziny (w punktach). Fig. 1. The average level of the meaning in life of lower secondary school students at the age of 13-17, in reference to satisfaction with life, subjective complaints and economic status of family.

## 2 Analiza wielowymiarowa dotycząca prawdopodobieństwa wysokiego poziomu sensu życia wśród uczniów gimnazjum

Do modelu regresji logistycznej włączono wszystkie analizowane zmienne, a także płeć i klasę gimnazjum. Kategoriami odniesienia były: brak zadowolenia z życia; często odczuwane dolegliwości fizyczne; rodzina biedna; III klasa gimnazjum i płeć − dziewczęta. Analizy wykazały, że zadowolenie z życia, odczuwanie dolegliwości fizycznych rzadko i z przeciętną częstotliwością oraz co najmniej przeciętny status ekonomiczny rodziny są związane z wysokim poziomem sensu życia (tab. III). Stwierdzono, że zadowolenie z życia blisko pięciokrotnie zwiększało prawdopodobieństwo wysokiego poziomu sensu życia [OR=4,70], w porównaniu z uczniami niezadowolonymi. Ponad dwukrotnie większe prawdopodobieństwo wysokiego poziomu sensu życia mieli uczniowie gimnazjum, którzy rzadko odczuwali fizyczne dolegliwości [OR=2,46], a 1,5-krotnie większe − uczniowie deklarujący występowanie dolegliwości z przeciętną częstotliwością [OR=1,51], w porównaniu z uczniami skarżącymi się na częste dolegliwości. Prawie 1,5-krotnie większe prawdopodobieństwo wysokiego poziomu sensu życia stwierdzono również wśród uczniów pochodzących z bogatych i przeciętnie zamożnych rodzin, w porównaniu z rodzinami biednymi.

**Tabela III:** Wyniki wielowymiarowej regresji logistycznej do oceny prawdopodobieństwa wysokiego poziomu sensu życia wśród uczniów gimnazjum. Table III. The results of multivariate logistic regression to assess the likelihood of a high level of the meaning in life among lower secondary school students.

Zmienne ** objaśniające *Explanatory variables*		Kategoria ** odniesienia *Reference category*	p	OR	95% CI (OR)
Zadowolenie z życia *Satisfaction with life*	Tak *Yes*	Nie *No*	**<0,001**	**4,70**	3,942-5,619
Dolegliwości fizyczne *Subjective physical complaints*	Rzadko odczuwane *Rarely felt*	Często odczuwane *Often felt*	<0,001	**2,46**	1,978-3,075
	Przeciętne odczuwane *On average felt*		**<0,001**	**1,50**	1,257-1,824
Status ekonomiczny rodziny *Economic status of family*	Raczej biedna *Rather poor*	Biedna *Poor*	0,053	1,26	0,997-1,595
	Przeciętna *Average*		**0,002**	**1,40**	1,131-1,747
	Bogata *Rich*		<0,001	**1,63**	1,275-2,092
Płeć *Sex*	Chłopcy *Boys*	Dziewczęta *Girls*	0,377	0,93	0,814-1,081
Klasa gimnazjum *Grade*	II *II* klasa *grade*	I klasa *Ist grade*	0,285	1,09	0,925-1,303
	III klasa *III grade*		0,429	0,93	0,790-1,105

## Omówienie

W niniejszej analizie posłużono się wynikami badania ankietowego przeprowadzonego na próbie 3695 uczniów w wieku 13-17 lat (I-III klasy gimnazjum). W pracy postawiono dwa pytania badawcze. Odpowiadając na pierwsze z nich, dotyczące różnicowania analizowanych zmiennych przez płeć i wiek, wykazano, że chłopcy istotnie częściej niż dziewczęta wykazywali się wyższym poziomem poczucia sensu życia. Rathi i Rastogi (2007) uzyskali w swych analizach inne wyniki [29]. W przeprowadzonym przez nich badaniu wśród nastolatków ustalili, że dziewczęta wykazały wyższe poczucie sensu życia niż chłopcy. Różnice w uzyskanych wynikach wynikać mogą z faktu, że autorzy zastosowali inne narzędzie badawcze – 57 – itemowy, siedmioczynnikowy *Personal Meaning Profile* (PMP). W przytoczonym badaniu wzięła także udział nieporównywalnie mniejsza próba nastolatków – 104 osoby. Nie bez znaczenia mogą tu także pozostawać różnice kulturowe. Chłopcy też istotnie bardziej niż dziewczęta byli zadowoleni z życia. Podobne wyniki uzyskano w badaniach HBSC 2014 [30] Badania Cömert i wsp. (2016) dotyczące m.in. zadowolenia z życia tureckich studentów nie potwierdzają jednak uzyskanych wyników, co może tłumaczyć fakt, iż autorzy poddali analizom młodych dorosłych, nie zaś nastolatków. [31] Odwrotne wyniki, dotyczące różnicowania zadowolenia z życia przez płeć uzyskali też Rathi i Rastogi (2007) [29]. W ich analizach to dziewczęta wykazały się wyższym poziomem satysfakcji z życia niż chłopcy. Autorzy użyli jednak do badania, nie Skali Cantrilla a Well-Being Manifestation Measure Scale (WBMMS). Częstość odczuwania przez młodzież dolegliwości subiektywnych była różnicowana istotnie statystycznie zarówno przez płeć, jak i klasę gimnazjum. Dziewczęta oraz uczniowie III klasy gimnazjum istotnie częściej niż chłopcy i uczniowie młodsi deklarowali nasilone odczuwanie dolegliwości subiektywnych. Uzyskane wyniki korespondują z rezultatami badań HBSC 2014, według których dziewczęta oraz uczniowie 15-letni wykazują się najczęstszymi dolegliwościami subiektywnymi [32]. Drugie pytanie badawcze dotyczyło zależności między zadowoleniem z życia, odczuwaniem dolegliwości somatycznych oraz statusu ekonomicznego rodziny a poczuciem sensu życia. Wykazano istotne statystycznie różnice w średnim poziomie sensu życia między uczniami w zależności od ich zadowolenia z życia i częstości odczuwania dolegliwości fizycznych oraz statusu ekonomicznego rodziny. Uczniowie zadowoleni ze swojego życia, rzadko odczuwający dolegliwości fizyczne oraz pochodzący z rodzin zamożnych, charakteryzowali się istotnie wyższym średnim poziomem sensu życia niż uczniowie, którzy byli niezadowoleni ze swojego życia, przeciętnie i często odczuwali dolegliwości oraz pochodzili z rodzin o najwyżej przeciętnym poziomie zamożności. Choć podobnych analiz dotyczących młodzieży szkolnej wśród innych autorów nie znaleziono, należy zwrócić uwagę, że doniesienia z badań HBSC przeprowadzonych w roku szkolnym 2013-2014 [30] wykazały, że zamożność rodziny wpływa na zadowolenie z życia nastolatków. Porównując rodziny najbiedniejsze i najzamożniejsze, autorzy stwierdzili wpływ zamożności we wszystkich analizowanych podgrupach wyróżnionych ze względu na płeć i wiek. Wyniki badania Tan i wsp. (2016) sugerują, że większe zadowolenie z życia wiąże się z wyższym poziomem uważności i bardziej pozytywną podstawową samooceną [33]. Uważność, jako szczególny rodzaj uwagi − świadomej nieosądzającej i skierowanej na bieżącą chwilę, może tu zostać powiązana z poczuciem sensu życia a pozytywna podstawowa samoocena wśród młodzieży często jest związana z zamożnością rodziny [34]. Międzykulturowe studia porównawcze Pan i wsp. (2008) przeprowadzone wśród chińskich studentów studiujących za granicą wskazują na sens życia jako czynnik ochronny satysfakcji z życia, w procesie akulturacji [35]. Wyniki wielu badań pokazują także, że sens życia ma istotny związek z dobrym samopoczuciem. Badania przeprowadzone w Australii przez Cohena i Cairns (2012) potwierdziły negatywny związek pomiędzy niskim poziomem sensu życia i subiektywnym samopoczuciem [36]. Halama i Dedova’ (2007) prowadząc badania wśród młodzieży słowackiej stwierdzili, że poczucie sensu życia jest mocnym predyktorem zadowolenia z życia [37]. Podobne wyniki uzyskano w badaniach przeprowadzonych wśród młodzieży w Hong Kongu, (Ho i wsp., 2010) [6]. Jeśli chodzi o związek poczucia sensu życia ze zdrowiem subiektywnym, to wcześniejsze analizy autorów niniejszego opracowania (Zawadzka i wsp., 2016) wykazały, że im lepiej gimnazjaliści oceniali swoje zdrowie, tym wyższym cechowali się poziomem sensu życia [24].

Biorąc pod uwagę związek poczucia sensu życia z zadowoleniem z życia, dolegliwościami subiektywnymi oraz statusem ekonomicznym rodziny, warto uwzględnić ten sposób myślenia w dalszych badaniach i działaniach z zakresu polityki zdrowotnej i edukacyjnej. Przede wszystkim należałoby wykorzystać potencjalny ochronny wpływ poczucia sensu życia na zdrowie, w programach promocji zdrowia, które powinny zawierać większy ładunek treści dotyczących zdrowia psychicznego, odporności psychicznej czy radzenia sobie ze stresem. W niektórych prywatnych lub liczących małą liczbę uczniów szkołach publicznych w Polsce prowadzone są spotkania mentoringowe a także tutoriale mające na celu poprawę dbałości młodzieży o zdrowie. W większych placówkach oświatowych warto wdrożyć serie spotkań socjoterapeutycznych w kilkunastoosobowych grupach lub klasach mających na celu naukę korzystania ze wsparcia społecznego. W Polsce brakuje aktualnych badań dotyczących sensu życia młodzieży szkolnej, dlatego celowe byłoby przeprowadzenie kolejnych analiz z uwzględnieniem innych zmiennych, takich jak: przemoc rówieśnicza czy subiektywna witalność a także porównań poziomu poczucia sensu życia u nastolatków zdrowych oraz z chorobami przewlekłymi w powiązaniu z innymi zmiennymi kontekstowymi.

Niewątpliwymi ograniczeniami przeprowadzonego badania było użycie w nim skróconych skal lub pojedynczych pytań dotyczących analizowanych zagadnień. Jest to charakterystyczne ograniczenie dotyczące dużych badań populacyjnych, które prowadzone są na kilkutysięcznych próbach, za pomocą wielowątkowych ankiet. Analizy te mają na celu, przede wszystkim informować o obecności pewnych zależnościach i wyznaczać kierunki dalszych, pogłębionych eksploracji. Należy jednak uwzględnić fakt, że są to pytania walidowane w badaniach międzynarodowych, dlatego rzetelność uzyskanych przy ich zastosowaniu wyników jest bardzo wysoka.

W niniejszym badaniu zastosowano również skróconą, 6-itemową wersję kwestionariusza PIL, opracowaną przez J. Życińską i J. Januszka [23]. Zdecydowano się na wykorzystanie skróconej wersji narzędzia, z obawy, że dla młodszych uczniów pełna wersja mogłaby się okazać zbyt trudna w percepcji i przez to też zostać pominięta podczas wypełniania kwestionariuszy.

Pomimo tego, niniejsza praca jest jednym z nielicznych opracowań obejmujących poczucie sensu życia młodzieży szkolnej. Zarówno ta, jak i wcześniejsze analizy, dotyczące tej problematyki (Zawadzka i wsp., 2016) [24], stanowią równocześnie punkt wyjścia do prowadzenia dalszych, planowanych już analiz.

## Wnioski

1. Według przeprowadzonych analiz, predyktorem wysokiego poziomu poczucia sensu życia wydaje się być zadowolenie z życia, dobre samopoczucie fizyczne oraz wyższa zamożność rodziny.
2. Skala PIL w wersji 6-punktowej jest godnym polecenia narzędziem w badaniach populacyjnych, przystępnym poznawczo również dla młodzieży szkolnej.
3. Ponieważ w literaturze polskiej brakuje analiz dotyczących poczucia sensu życia młodzieży szkolnej, warto kontynuować i pogłębiać badania, z uwzględnieniem zarówno skróconej, jak i pełnej wersji tego narzędzia.
